# Socio-cultural Correlates of the COVID-19 Outcomes

**DOI:** 10.1007/s44197-022-00055-3

**Published:** 2022-08-23

**Authors:** Timo Lajunen, Esma Gaygısız, Ümmügülsüm Gaygısız

**Affiliations:** 1 Department of Psychology, Norwegian University of Science and Technology (NTNU), Trondheim, Norway; 2 Department of Economics, Middle East Technical University, Ankara, Turkey; 3 Department of Anesthesia Intensive Care, Faculty of Medicine, Gazi University, 06560 Ankara, Turkey

**Keywords:** COVID-19 outcomes, Socio-economic factors, Hofstede's cultural dimensions, Mortality, Stringency index, GDP, Vaccination rate

## Abstract

While basically all countries have been hit by the COVID-19 pandemic, the impact has varied in large degrees among countries. In the present study, national differences in six COVID-19 indicators (COVID-19 deaths per capita, excess mortality, change in GDP per capita, vaccination rate, stringency index, and overall impact of the pandemic) were studied in relation to socio-economic and Hofstede's cultural dimensions by using the latest data available. The results differed to some degree from the studies conducted in the earlier stage of the pandemic. COVID-19 deaths per capita were predicted by Uncertainty Avoidance (UA) and Indulgence (IVR); excess mortality by UA; the impact of pandemics by Power Distance (PDI), Long-term Orientation (LTOWS) and IVR; change in GDP per capita by PDI; vaccination rate by Individualism and UA; and Stringency Index by LTOWS. In addition to further clarifying the role of cultural dimensions in the pandemic, three conclusions can be drawn. First, the pandemic reached different countries at different times, which is reflected in the results. The conclusion about the role of socio-economic and cultural factors can be drawn only after the pandemic. Second, cultural dimensions were related to COVID-19 measures only when socio-economic indicators were not considered but lost their significance when socio-economic variables were entered into the models. Cultural dimensions influence the outcome variables via socio-economic factors. Third, earlier studies have focused mainly on COVID-19 deaths. The impact of the COVID-19 pandemic is a complex phenomenon and cannot be reduced to the death rate.

## Introduction

Up to date (23 July 2021), the COVID-19 pandemic has claimed 4,136,518 lives globally. The highest number of deaths has been reported in the USA (604,546 deaths), followed by Brazil (545,604 deaths) and India (419,470 deaths) [1]. In terms of fatalities per million people, the highest COVID-19 stricken country is Peru, with 5921.51 deaths per million people, followed by Hungary (3107.55 deaths per million) and Bosnia and Herzegovina (2948.96 deaths per million) [2]. Closer inspection of COVID-19 databases shows a vast heterogeneity among countries and regions within countries in terms of confirmed cases and deaths per capita as well as in terms of infection fatality and vaccination rates.

Several studies have investigated possible socio-economic or population factors which could explain the regional differences in COVID-19 outcomes. The most investigated economic factors seem to be GPD per capita, income inequality measured with the Gini index, and health care expenses per capita. In several studies, the GDP per capita has been positively related to higher COVID-19 death rates [3–9] while in some other studies no relationship between GDP per capita and COVID-19 deaths has been reported [10]. Asfahan et al. (2020) found a negative relationship between GDP and case fatality rate [11]. One study reported a positive relationship between GPD and COVID-19 deaths on 14 July 2020, but not on 29 December 2020 [12], indicating that the GDP—COVID-19 death relationship depends on the phase of the pandemic. Also, the set of countries included in the analysis seems to make a difference: Cifuentes-Faura reported a positive relationship between GDP and COVID-19 deaths in Latin American countries [13]. The positive correlation between GDP per capita and COVID-19 mortality can be explained by higher mobility and level of economic activity. On the other hand, high GDP per capita might correlate with higher expenditure on public health services and resources available, which in turn are negatively related to COVID-19 mortality [11, 13, 14]. Income inequality has attracted much less interest than GDP among researchers. In their 50-country study, Chaudhry et al. (2020) found that reduced income dispersion reduced COVID-19 mortality. Similarly, Wildman (2021) demonstrated that the OECD countries with high levels of income inequality have performed significantly worse when dealing with the COVID-19 outbreak in terms of cases and deaths compared to countries with low inequality. In studies based on US counties, income inequality was related to higher mortality [15, 16]. As Wildman suggests, income inequality is a proxy for many elements of socio-economic disadvantages, such as inadequate housing, smoking, obesity, and pollution, which can increase COVID-19 mortality [17].

Such population factors as high median age, obesity and population density can be expected to be related to higher COVID-19 mortality because COVID-19 infection seems to be especially risky for elderly patients and patients with obesity or chronic illnesses such as Type 2 diabetes mellitus, hypertension, and other metabolic comorbidities [18, 19]. The relationship between obesity (BMI ≥ 30) and COVID-19 mortality has been demonstrated in several studies [3, 8, 9, 12]. Moreover, the higher median age of the population seems to increase COVID-19 mortality [3, 9, 11, 20–22]. The third population variable often included in the studies is population density. It can be assumed that the SARS-CoV-2 virus spreads quickly in dense populations as any other infectious disease [23, 24], which was indeed reported in the study by Erman and Medeiros (2021). The relationship between population density or urbanization rate and COVID-19 deaths seems, however, to be somewhat unclear. In one study including data from 37 countries, COVID-19 deaths were positively associated with population density [22], which has also been found in some other studies [25], while in some studies population density has had no relationship to COVID-19 mortality [12]. In some studies, the urbanization rate has had a stronger relationship to COVID-19 mortality than population density [26]. On the other hand, a study including only Latin American countries found that countries with higher population density had a lower number of deaths [13]. One reason for these somewhat mixed results might be that in low population density (rural) regions, people have fewer contacts with each other compared to densely populated areas, while the health services are usually better and more available in urban areas. This might explain why the crude infection rate is higher and mortality lower in unurbanized areas [10]. If a country provides equally good health care services in all regions regardless of the population density, we could expect both infection rate and mortality to be lower in less densely populated areas.

A pandemic is a crisis that requires effective crisis management to mitigate the damage and secure the functioning of society. For example, countries have to decide about policies related to facial masks, social distancing, screening travellers, guaranteeing symptomatic patients, contact tracing and vaccination priorities. Curfews, closedown of businesses and obligatory distance learning at schools are all harsh measures in which the authorities must balance health risks and harm done to societal life. The effectiveness of countries' response to the COVID-19 pandemic might reflect the general quality of governance in general. In some studies, good governance has been found to be related to fewer COVID-19 deaths [27–30], while in some other studies the results have been inconclusive [12]. In the present study, we measured governance Worldwide Governance Indicators (WGI) published by World Bank [31]. WGI consists of the following six dimensions of governance: Voice and Accountability, Political Stability and Absence of Violence, Government Effectiveness, Regulatory Quality, Rule of Law‖, and Control of Corruption. A single merged index for the WGI was used in the present study [32].

While socio-economic factors, population characteristics and governance quality certainly are important factors influencing, it should be noted that many of these factors reflect national cultural values. For example, policies for reducing income equality, investment in the public health care sector and, finally, restrictions on citizens' activities or business are all political decisions that are based on specific values. Therefore, it is not surprising that some social scientists have investigated the role of cultural values using such theories as Schwartz’s Basic Human Values [12, 33, 34] or Hofstede’s dimensions of culture [12, 21, 35–39]. In the present study, the relationship between different COVID-19 outcome measures and Hofstede’s dimensions of culture was studied.

Culture can be called “the collective programming of the mind that distinguishes the members of one group or category of people from another” [40]. The centre of the mechanism of culture is “a system of societal norms consisting of the value systems (or the mental software) shared by major groups in the population”. Hofstede (2001) represented the fundamental problems of societies by investigating culture through originally four and later six empirically identified dimensions. These dimensions were inequality between people (PDI: power distance), the level of stress in a society related to the unknown future (UAI: uncertainty avoidance), the integration of individuals into primary groups (IDV: Individualism vs collectivism), the division of emotional roles between men and women (MAS: masculinity vs femininity), how the culture deals with change (LTOVWS: Long-Term Orientation vs Short-Term Orientation) and how much the society allows relatively free gratification of natural human desires related to enjoying life and having fun [41]. In previous studies, especially Individualism has been positively related to COVID-19 mortality [10, 12, 34, 38]. Besides, in some studies, uncertainty avoidance has been negatively related to COVID-19 mortality [38]. In some other studies, it has had a positive relationship with mortalities per capita [10]. In one study, it was reported that Uncertainty Avoidance predicts a lower proportion of people gathering in public [37].

Almost all the studies use fatalities per capita, infections per capita, or fatalities per detected infections as a criterion (dependent or predicted variable) in analyses. These measures are, however, challenging in many ways. First, the reported numbers of cases and deaths can be strongly affected by testing capacity and reporting policy, resulting in significant underreporting [42]. This underreporting can systematically bias the analyses showing countries with less testing capacity better in terms of COVID-19 mortality. In one study, the excess mortality was above 50% of the expected annual providing a more reliable estimate than COVID-19 deaths per infection or capita [42]. It is also possible that the pandemic and related restrictions have influenced the data collection routines used in collecting annual socio-economic data. More importantly, this bias might have changed according to the stage of the pandemic and the related restrictions. For example, face-to-face interviews about corruption can lead to different results than internet-based interviews. Second, the pandemic has reached different countries in different time frames. Since most of the data in recent studies were collected in 2020, we should consider it as historical reflecting the situation in that current moment. Today the COVID-19 situation is much different in many countries, and the situation in many countries has improved considerably (e.g., the UK, Sweden). Due to the fast vaccine rollout and improved COVID-19 situation, many countries have lifted most of the restrictions, which is likely to influence people’s lifestyles and daily activities leading to change in socio-economic indicators too. Third, mortality per capita gives a narrow view of the total national cost of the COVID-19 pandemic. In the present study, the impact of the COVID-19 pandemic was measured with six performance and impact measures: COVID-19 deaths per 100,000 people, a composite score of COVID-19 pandemic performance, change in GDP per capita, excess mortality, vaccination percentage, and stringency index. In this way, different aspects of the pandemic were used as the predicted (dependent) variable, giving a complete picture of the national differences.

The present study aimed to investigate how socio-economic and cultural factors are related to six national COVID-19 pandemic outcomes and performance measures.

## Materials and Methods

The data were downloaded from various online sources. The data included six COVID-19 related dependent variables (variables 1–6 in Table 1), socio-economic independent variables (predictors) (variables 7–16 in Table 1) and Hofstede’s cultural dimensions (variables 17–22).

**Table 1:** Descriptions of the variables included in the study

Variable	References
1. COVID-19 deaths per 100,000 people on 21 July 2021	[43]
2. Change (%) in Gross Domestic Product (GDP) for 2020	[44]
3. Excess mortality from 1 January 2020 to 10 May 2021	[45]
4. Vaccination rate (at least one vaccination) of the population (%) on 30 June 2021	[43]
5. Stringency index, measuring ‘lockdown style’ policies that restrict people’s behaviour	[46]
6. Overall score of the pandemic impact (average of variables 2, 3, 4, and 5)	[47]
7. GDP per capita 2019	[48]
8. Gini index 2010–2018	[48]
9. Urbanization rate (%)	[48]
10. WGI	[49]
11. Life expectancy	[48]
12. Years of schooling	[48]
13. Median age	[48]
14. Prevalence of obesity (BMI ≥ 30) among adults (%)	[50]
15. Healthcare expenditure	[48]
16. Physicians per 10,000 people	[48]
17. Power Distance	[41]
18. Individualism—Collectivism	[41]
19. Masculinity—Femininity	[41]
20. Uncertainty—Avoidance	[41]
21. Short—Long-term Orientation	[41]
22. Indulgence—Restraint	[41]

The number of countries included in correlation analyses varied between 65 and 153 countries, whereas the number of countries included in regression analysis was 52.

## Results

### Correlations Between the COVID-19 Pandemic Indicators and Socio-Cultural Variables

Correlations between COVID-19 measures and socio-economic and cultural dimensions can be seen in Table 2. COVID-19 deaths per 100,000 people correlated with excess mortality (*r* = 0.64), which shows that excess mortality is due to COVID-19 deaths to a great degree. However, the COVID-19 deaths do not account for the variance of the excess mortality of more than 41.0%, which means that excess mortality also captures those deaths which might be indirectly related to SARS-CoV-2 infection, e.g., fatalities caused by the conditions related to pandemic (e.g., postponed medical operations, suicides).

**Table 2:** Correlations between the COVID-19 outcome variables and socio-cultural variables

	1	2	3	4	5	6
1. Deaths per 100,000 people	1.00					
2. Overall impact of COVID-19 pandemics	0.28***	1.00				
3. Change in GDP per capita	– 0.15	– 0.37***	1.00			
4. Excess mortality	0.64***	0.59***	0.00	1.00		
5. Vaccination percentage	0.39***	– 0.45***	– 0.06	– 0.06	1.00	
6. Stringency Index	0.31***	0.51***	– 0.18*	0.15	0.26***	1.00
7. GDP per capita 2019	0.27***	– 0.51***	0.07	– 0.13	0.76***	0.12
8. Gini index	– 0.08	0.29***	– 0.08	0.06	– 0.36***	0.02
9. Urbanization	0.40***	– 0.19*	– 0.10	0.06	0.63***	0.27***
10. WGI	0.33***	– 0.51***	0.04	– 0.20*	0.74***	0.07
11. Life expectancy	0.46***	– 0.21**	– 0.07	0.05	0.72***	0.33***
12. Years of schooling	0.47***	– 0.20*	– 0.01	0.12	0.62***	0.23**
13. Median age	0.53***	– 0.25**	– 0.02	0.15	0.72***	0.21*
14. Obesity (%)	0.50**	0.01	– 0.21*	0.30***	0.57***	0.27***
15. Healthcare expenditure	0.35***	– 0.20*	0.03	0.08	0.37***	– 0.03
16. Physicians	0.49***	– 0.21**	– 0.01	0.19*	0.64***	0.17*
17. Power Distance	0.10	0.64***	– 0.37**	0.40***	– 0.45***	0.24
18. Individualism	0.05	– 0.50***	0.19	– 0.17	0.58***	– 0.22
19. Masculinity	0.11	0.18	– 0.11	0.09	– 0.07	0.15
20. Uncertainty Avoidance	0.39***	0.21	– 0.19	0.42***	0.15	– 0.02
21. Long-term Orientation	0.16	– 0.24*	0.13	0.09	0.23*	– 0.32**
22. Indulgence	– 0.01	– 0.29**	– 0.01	– 0.30**	0.27*	0.02

*p ≤ 0.05; **p ≤ 0.01; ***p ≤ 0.001

Comparison of correlations between COVID-19 deaths and socio-cultural variables with excess mortality per 100,000 inhabitants and socio-cultural variables show that COVID-19 deaths correlated significantly with most of the socio-cultural variables, whereas excess mortality correlated statistically significantly only with WGI, obesity, physicians per 10,000 people, Power Distance, Uncertainty Avoidance, and Indulgence. Interestingly, the only cultural dimensions which COVID-19 deaths correlated significantly with was Uncertainty Avoidance.

The overall score of COVID-19 impact composed of change in GDP, excess mortality, vaccination percentage and stringency index correlated relatively weakly but significantly (*r* = 0.28) with COVID-19 deaths per capita. Bearing in mind that a high total score means a high (negative) impact of the pandemic, in this model, COVID-19 deaths seemed to be only one aspect in the total view of how the pandemic has impacted a country. Unlike the death rate having a negative correlation to GDP per capita before the pandemic, the total score correlated positively with GDP. The strongest correlations among the total impact score and socio-cultural variables were between the total score and GDP per capita 2019 (*r* = − 0.51), WGI (*r* = − 0.51), Power Distance (*r* = 0.64), and Individualism (*r* = − 0.50). These correlations indicate that high-income individualistic countries with high-quality governance but low Power Distance have been less impacted by the pandemic than the other countries. It should be noted that the COVID-19 deaths per capita have a positive correlation to GDP and WGI but non-significant correlations to Individualism and Power Distance while correlating positively with Uncertainty Avoidance.

### The Hierarchical Regression Analysis Results

A total of six hierarchical regression analyses were conducted for the COVID-19 variables (variables 1–6 listed in Table 2). The study variables were entered into the model in two blocks: Hofstede’s cultural dimensions (variables 17–22 listed in Table 2) in the first block and the added socio-economic variables (variables 7–16 listed in Table 2) together with Hofstede’s cultural values in the second. The variables were entered in this order because we assumed that cultural values influence all behaviours and socio-economic variables in the background. If the socio-economic variables—the consequences of culture—are controlled in the first step, the effects of cultural variables are likely to disappear. This can be seen in Table 3: in every analysis, the cultural dimensions lost their significance when socio-economic variables were entered into the model. The results for six hierarchical regression analyses can be found in Table 3.

**Table 3:** Hierarchical regression analysis results

Block	Variables	*B*	SE B	*β*	*t*	95% CI LB	95% CI UB	*R*^2^
Dependent Variable: Deaths per 100,000 people								
1	(Constant)	– 81.40	120.62		– 0.68	– 324.33	161.54	0.18
	Power Distance	0.31	0.99	0.06	0.31	– 1.68	2.30	
	Individualism	0.95	0.91	0.19	1.04	– 0.89	2.79	
	Masculinity	0.37	0.75	0.07	0.49	– 1.15	1.89	
	Uncertainty Avoidance	2.15	0.73	0.42	2.96**	0.69	3.62	
	Long-term Orientation	– 0.44	0.80	– 0.09	– 0.55	– 2.06	1.18	
	Indulgence	0.00	0.86	0.00	0.01**	– 1.72	1.73	
2	(Constant)	– 49.69	710.98	0.00	– 0.07	– 1493.05	1393.67	0.33
	Power Distance	– 0.17	1.20	– 0.03	– 0.14	– 2.60	2.26	
	Individualism	– 0.92	1.56	– 0.19	– 0.59	– 4.08	2.25	
	Masculinity	1.02	0.94	0.20	1.09	– 0.88	2.92	
	Uncertainty Avoidance	1.12	1.32	0.22	0.85	– 1.56	3.80	
	Long-term Orientation	– 0.28	1.29	– 0.06	– 0.22	– 2.89	2.34	
	Indulgence	– 0.57	1.32	– 0.11	– 0.43	– 3.25	2.11	
	GDP per capita 2019	0.00	0.00	– 0.16	– 0.54	0.00	0.00	
	Gini index	3.24	3.91	0.19	0.83	– 4.70	11.18	
	Urbanization	0.26	1.99	0.04	0.13	– 3.79	4.30	
	WGI	23.02	51.36	0.17	0.45	– 81.25	127.29	
	Life expectancy	– 3.32	10.41	– 0.12	– 0.32	– 24.46	17.82	
	Years of schooling	14.82	15.35	0.31	0.97	– 16.35	45.99	
	Median age	1.01	7.07	0.06	0.14	– 13.34	15.36	
	Obesity (%)	4.86	3.73	0.38	1.30	– 2.71	12.43	
	Healthcare expenditure	– 1.44	9.41	– 0.04	– 0.15	– 20.54	17.66	
	Physicians	– 0.30	2.03	– 0.04	– 0.15	– 4.43	3.82	
Dependent Variable: Overall impact of COVID-19 pandemics								
1	(Constant)	3.48	0.62		5.64	2.24	4.72	0.58
	Power Distance	0.02	0.01	0.41	3.07**	0.01	0.03	
	Individualism	– 0.01	0.01	– 0.21	– 1.59	– 0.02	0.00	
	Masculinity	0.01	0.00	0.21	1.97	0.00	0.02	
	Uncertainty Avoidance	0.00	0.00	0.01	0.04	– 0.01	0.01	
	Long-term Orientation	– 0.01	0.00	– 0.39	– 3.29**	– 0.02	– 0.01	
	Indulgence	– 0.01	0.00	– 0.25	– 2.07*	– 0.02	0.00	
2	(Constant)	2.78	3.16	0.00	0.88	– 3.63	9.19	0.74
	Power Distance	0.01	0.01	0.22	1.56	0.00	0.02	
	Individualism	0.01	0.01	0.15	0.78	– 0.01	0.02	
	Masculinity	0.00	0.00	0.09	0.76	– 0.01	0.01	
	Uncertainty Avoidance	0.01	0.01	0.19	1.15	– 0.01	0.02	
	Long-term Orientation	0.00	0.01	– 0.09	– 0.57	– 0.02	0.01	
	Indulgence	0.00	0.01	0.11	0.70	– 0.01	0.02	
	GDP per capita 2019	0.00	0.00	– 0.29	– 1.56	0.00	0.00	
	Gini index	0.03	0.02	0.21	1.48	– 0.01	0.06	
	Urbanization	– 0.02	0.01	– 0.34	– 1.86	– 0.03	0.00	
	WGI	– 0.25	0.23	– 0.26	– 1.11	– 0.72	0.21	
	Life expectancy	0.00	0.05	– 0.02	– 0.07	– 0.10	0.09	
	Years of schooling	0.08	0.07	0.24	1.17	– 0.06	0.22	
	Median age	– 0.02	0.03	– 0.16	– 0.59	– 0.08	0.05	
	Obesity (%)	0.00	0.02	0.01	0.05	– 0.03	0.03	
	Healthcare expenditure	– 0.02	0.04	– 0.09	– 0.57	– 0.11	0.06	
	Physicians	0.01	0.01	0.12	0.75	– 0.01	0.03	
Dependent variable: change in GDP per capita								
1	(Constant)	0.51	3.69		0.14	– 6.93	7.95	0.23
	Power Distance	– 0.07	0.03	– 0.39	– 2.20*	– 0.13	– 0.01	
	Individualism	– 0.02	0.03	– 0.11	– 0.61	– 0.07	0.04	
	Masculinity	– 0.01	0.02	– 0.06	– 0.45	– 0.06	0.04	
	Uncertainty Avoidance	– 0.04	0.02	– 0.22	– 1.61	– 0.08	0.01	
	Long-term Orientation	0.03	0.03	0.16	1.02	– 0.03	0.08	
	Indulgence	– 0.02	0.03	– 0.14	– 0.85	– 0.08	0.03	
2	(Constant)	– 17.64	19.26	0.00	– 0.92	– 56.74	21.46	0.51
	Power Distance	– 0.07	0.03	– 0.38	– 1.99	– 0.13	0.00	
	Individualism	– 0.06	0.04	– 0.37	– 1.35	– 0.14	0.03	
	Masculinity	– 0.02	0.03	– 0.13	– 0.84	– 0.07	0.03	
	Uncertainty Avoidance	– 0.07	0.04	– 0.40	– 1.82	– 0.14	0.01	
	Long-term Orientation	0.04	0.04	0.25	1.11	– 0.03	0.11	
	Indulgence	– 0.06	0.04	– 0.38	– 1.74	– 0.14	0.01	
	GDP per capita 2019	0.00	0.00	0.55	2.12*	0.00	0.00	
	Gini index	– 0.11	0.11	– 0.21	– 1.08	– 0.33	0.10	
	Urbanization	0.04	0.05	0.18	0.71	– 0.07	0.15	
	WGI	– 4.4	1.39	– 1.03	– 3.16**	– 7.22	– 1.57	
	Life expectancy	0.32	0.28	0.38	1.14	– 0.25	0.90	
	Years of schooling	0.43	0.42	0.28	1.02	– 0.42	1.27	
	Median age	– 0.25	0.19	– 0.48	– 1.32	– 0.64	0.14	
	Obesity (%)	0.06	0.10	0.14	0.56	– 0.15	0.26	
	Healthcare expenditure	0.33	0.26	0.27	1.30	– 0.19	0.85	
	Physicians	0.02	0.06	0.07	0.31	– 0.10	0.13	
Dependent variable: excess mortality								
1	(Constant)	– 27.28	135.33		– 0.20	– 299.84	245.28	0.28
	Power Distance	1.75	1.11	0.27	1.58	– 0.49	3.98	
	Individualism	0.68	1.03	0.12	0.67	– 1.38	2.75	
	Masculinity	0.30	0.85	0.05	0.36	– 1.40	2.00	
	Uncertainty Avoidance	2.09	0.82	0.35	2.57*	0.45	3.74	
	Long-term Orientation	– 0.83	0.90	– 0.14	– 0.93	– 2.65	0.98	
	Indulgence	– 1.54	0.96	– 0.25	– 1.61	– 3.47	0.39	
2	(Constant)	831.93	657.27	0.00	1.27	– 502.41	2166.26	0.60
	Power Distance	0.47	1.11	0.07	0.42	– 1.78	2.71	
	Individualism	– 0.57	1.44	– 0.10	– 0.39	– 3.49	2.36	
	Masculinity	0.55	0.86	0.09	0.64	– 1.20	2.31	
	Uncertainty Avoidance	1.29	1.22	0.21	1.06	– 1.18	3.77	
	Long-term Orientation	0.82	1.19	0.14	0.69	– 1.60	3.24	
	Indulgence	0.18	1.22	0.03	0.14	– 2.31	2.66	
	GDP per capita 2019	0.00	0.00	– 0.06	– 0.24	0.00	0.00	
	Gini index	0.78	3.61	0.04	0.22	– 6.55	8.12	
	Urbanization	– 1.67	1.84	– 0.21	– 0.91	– 5.42	2.07	
	WGI	– 41.69	47.48	– 0.26	– 0.88	– 138.09	54.7	
	Life expectancy	– 14.18	9.63	– 0.44	– 1.47	– 33.72	5.37	
	Years of schooling	12.48	14.19	0.22	0.88	– 16.34	41.29	
	Median age	1.37	6.54	0.07	0.21	– 11.89	14.64	
	Obesity (%)	7.89	3.45	0.52	2.29*	0.89	14.89	
	Healthcare expenditure	1.26	8.70	0.03	0.15	– 16.4	18.92	
	Physicians	0.98	1.88	0.11	0.52	– 2.84	4.79	
Dependent variable: vaccination percentage								
1	(Constant)	– 18.48	14.57		– 1.27	– 47.83	10.87	0.63
	Power Distance	– 0.13	0.12	– 0.13	– 1.07	– 0.37	0.11	
	Individualism	0.54	0.11	0.61	4.88***	0.32	0.76	
	Masculinity	– 0.14	0.09	– 0.15	– 1.59	– 0.33	0.04	
	Uncertainty Avoidance	0.40	0.09	0.44	4.52***	0.22	0.58	
	Long-term Orientation	0.11	0.10	0.12	1.13	– 0.09	0.31	
	Indulgence	0.26	0.10	0.28	2.54*	0.05	0.47	
2	(Constant)	25.57	71.35	0.00	0.36	– 119.29	170.42	0.79
	Power Distance	0.02	0.12	0.02	0.13	– 0.23	0.26	
	Individualism	0.13	0.16	0.14	0.80	– 0.19	0.44	
	Masculinity	0.00	0.09	0.00	0.03	– 0.19	0.19	
	Uncertainty Avoidance	0.16	0.13	0.17	1.19	– 0.11	0.43	
	Long-term Orientation	0.11	0.13	0.12	0.85	– 0.15	0.37	
	Indulgence	0.08	0.13	0.09	0.61	– 0.19	0.35	
	GDP per capita 2019	0.00	0.00	0.00	0.02	0.00	0.00	
	Gini index	– 0.32	0.39	– 0.10	– 0.80	– 1.11	0.48	
	Urbanization	– 0.02	0.20	– 0.02	– 0.10	– 0.43	0.39	
	WGI	16.18	5.16	0.67	3.14**	5.71	26.64	
	Life expectancy	– 0.08	1.05	– 0.02	– 0.08	– 2.2	2.04	
	Years of schooling	– 3.87	1.54	– 0.45	– 2.51*	– 7.00	– 0.74	
	Median age	0.24	0.71	0.08	0.34	– 1.20	1.68	
	Obesity (%)	1.03	0.37	0.44	2.74**	0.27	1.79	
	Healthcare expenditure	0.61	0.94	0.09	0.65	– 1.30	2.53	
	Physicians	0.00	0.20	0.00	– 0.01	– 0.42	0.41	
Dependent variable: stringency index								
1	(Constant)	0.03	8.02		8.33	50.63	82.93	0.37
	Power Distance	– 0.06	0.07	0.08	0.47	– 0.10	0.16	
	Individualism	0.10	0.06	– 0.16	– 0.97	– 0.18	0.06	
	Masculinity	– 0.05	0.05	0.24	1.91	– 0.01	0.20	
	Uncertainty Avoidance	– 0.20	0.05	– 0.14	– 1.08	– 0.15	0.05	
	Long-term Orientation	– 0.03	0.05	– 0.53	– 3.72***	– 0.31	– 0.09	
	Indulgence	– 55.90	0.06	– 0.07	– 0.45	– 0.14	0.09	
2	(Constant)	0.03	43.11	0.00	– 1.30	– 143.42	31.63	0.57
	Power Distance	0.12	0.07	0.08	0.47	– 0.11	0.18	
	Individualism	0.01	0.09	0.33	1.31	– 0.07	0.32	
	Masculinity	0.04	0.06	0.02	0.11	– 0.11	0.12	
	Uncertainty Avoidance	– 0.05	0.08	0.10	0.49	– 0.12	0.20	
	Long-term Orientation	– 0.03	0.08	– 0.14	– 0.65	– 0.21	0.11	
	Indulgence	0.00	0.08	– 0.07	– 0.34	– 0.19	0.14	
	GDP per capita 2019	0.44	0.00	– 0.26	– 1.09	0.00	0.00	
	Gini index	– 0.24	0.24	0.33	1.84	– 0.05	0.92	
	Urbanization	– 1.87	0.12	– 0.46	– 1.95	– 0.48	0.01	
	WGI	1.92	3.12	– 0.18	– 0.60	– 8.19	4.45	
	Life expectancy	0.37	0.63	0.95	3.04**	0.64	3.20	
	Years of schooling	– 1.21	0.93	0.10	0.40	– 1.52	2.26	
	Median age	– 0.12	0.43	– 0.97	– 2.83**	– 2.08	– 0.34	
	Obesity (%)	0.05	0.23	– 0.12	– 0.51	– 0.58	0.34	
	Healthcare expenditure	0.17	0.57	0.02	0.08	– 1.11	1.21	
	Physicians	0.03	0.12	0.28	1.34	– 0.09	0.42	

In the first analysis, COVID-19 deaths per 100,000 people were regressed first to cultural dimensions (Block 1) and then to socio-economic variables (Block 2). Hofstede's cultural dimensions accounted for 18% of the variance. In Block 1, Uncertainty Avoidance and Indulgence were positively related to COVID-19 deaths. The socio-economic variables accounted for an added share of 15% of the variance, but none of the individual variables had a significant effect on the deaths per 100,000 people.

In the second analysis, the overall impact of the COVID-19 pandemic was regressed to cultural dimensions (Block 1) and then to socio-economic variables (Block 2). In Block 1 (58% of the variance accounted for), Power Distance was positively, and Uncertainty Avoidance and Long-Term Orientation were negatively related to the overall COVID-19 impact score. Since a low overall score means a low (negative) impact of the pandemic, we can conclude that countries with high Power Distance and low Uncertainty Avoidance and low Short-Term Orientation were more harmed by the pandemic. The socio-economic variables accounted for an added share of 16% of the variance, but none of the individual variables had a significant effect on the overall score.

In the third analysis, the change in GDP per capita during the COVID-19 pandemic was regressed to cultural dimensions (Block 1) and then to socio-economic variables (Block 2). In Block 1 (23% of the variance accounted for), only Power Distance was related to the change in GDP per capita. In GDP change, a positive number means an increase in GDP and a negative number a decrease in GDP per capita, so the COVID-19 pandemic seemed to have a larger (negative) effect on the economy (measured with GDP) when a country scored high in Power Distance. However, it should be noted that only Iran and Taiwan showed an increase in GDP per capita during the pandemic while all the GDP of the other countries shrank to smaller or larger degrees. The socio-economic variables accounted for an added share of 28% of the variance, but none of the individual variables had a significant effect on the change in GDP.

In the fourth analysis, excess mortality was regressed to cultural dimensions (Block 1) and then to socio-economic variables (Block 2). In Block 1 (28% of the variance accounted for), only Uncertainty Avoidance had a statistically significant relationship to excess mortality. Countries with higher Uncertainty Avoidance scores had higher excess mortality during 2020. The socio-economic variables accounted for an added share of 32% of the variance. Among the socio-economic variables, the obesity rate was positively related to excess mortality.

In the fifth analysis, the percentage of vaccinated (at least one vaccination) was regressed to cultural dimensions (Block 1) and then to socio-economic variables (Block 2). In Block 1 (63% of the variance accounted for), Individualism and Uncertainty Avoidance had a statistically significant relationship to vaccination rate. Countries with higher Uncertainty Avoidance and Individualism score had higher vaccination rates. The socio-economic variables accounted for an added share of 16% of the variance. Among the socio-economic variables, WGI, years of schooling and obesity rate were positively related to the proportion of the population vaccinated against the SARS-CoV-2 virus.

In the last regression analysis, the stringency index was regressed to cultural dimensions (Block 1) and then to socio-economic variables (Block 2). In Block 1 (37% of the variance accounted for), only Long-Term Orientation had a statistically significant relationship to vaccination rate. Countries with a higher score in Long-Term Orientation applied less stringent COVID-19 policies. The socio-economic variables accounted for an added share of 20% of the variance. Among the socio-economic variables, life expectancy was positively, and the median age was negatively related to stringent policies.

## Discussion

Since the beginning, the COVID-19 pandemic has cost more than four million lives. While being the most painful outcome of the pandemic, COVID-19 deaths are just one of the many adverse outcomes of the pandemic. Even SARS-CoV-2 infection with mild or unnoticeable symptoms may lead to "long COVID" characterized by long-lasting fatigue, cough, chest tightness, headaches, breathlessness, palpitations, myalgia and difficulty to focus [51]. It has also been reported that depressive, anxiety and post-traumatic symptoms may result from SARS-CoV-2 infection [52]. A registry-based study in Sweden showed that a substantial number of people have been on sick leave due to COVID-19. Sick leave was often prolonged, and sick leave for long-COVID was relatively common [53]. In addition to this vast impact on public health, the COVID-19 pandemic has had a profound negative effect on economies, including decreased GDP for 2020 [54], businesses [55] and unemployment [56].

Countries differ in a large degree in terms of their "resilience" to the effects of the COVID-19 pandemic. Research has indicated that certain socio-cultural and economic factors make countries more or less impacted by the COVID-19 pandemic. In addition to economic, political, and population-related factors, several studies have investigated the role of cultural values in the effects of the COVID-19 pandemic. One of the most robust findings is that Individualism (as Hofstede's cultural dimension) is positively related to COVID-19 deaths [10, 12, 21, 34, 38, 57]. Besides, Individualism has been found to be positively related to a higher prevalence rate and fatality rate among infected [10, 21]. The results of the present study differed clearly from those found earlier: Hofstede’s Individualism dimension did not correlate significantly (*r* = 0.05) with COVID-19 deaths per capita, and the excess mortality rate correlated negatively (*r* = − 0.17) with COVID-19 mortality per capita. These findings do not support the claim by Grüss and Tusaon (2021) that «Individualism…can kill». In fact, Individualism correlated negatively with the overall COVID-19 impact score, indicating that individualistic countries have been less impacted by the COVID-19 pandemic than collectivistic countries. Vaccination percentage, for example, had a strong correlation (*r* = 0.58) with Individualism and Stringency Index negative (*r* = − 0.22, n.s.) correlation with Individualism. The explanation for the difference between the findings of the current and earlier studies might be straightforward: earlier studies naturally applied data from the earlier stages of the pandemic, while the present study is based on the most recent data. The COVID-19 pandemic has spread to different countries at a different speed, Europe having been impacted heavily in a very early stage of the pandemic. Besides, wealthy individualistic countries are leading in the current vaccination percentage, which naturally is reflected in COVID-19 mortality. Hence, the results of the earlier studies and the present study do not conflict: they reflect the different phases of the pandemic (e.g., pre-vaccination vs post-vaccination).

In addition to Individualism, also Uncertainty Avoidance has appeared to be an important factor in relation to COVID-19 deaths, although the findings have been somewhat conflicting. Reman and Medeiros (2021) found a positive relationship between Uncertainty Avoidance and COVID-19 deaths and infection rate. In Oey & Rahardjo (2021), Uncertainty Avoidance was negatively related to COVID-19 deaths. Hunyh (2020) reported that Uncertainty Avoidance predicted the lower proportion of people gathering in public such as retail and recreation, grocery and pharmacy, parks, transit stations, and workplaces. In our study, Uncertainty Avoidance correlated with COVID-19 deaths per 100,000 people (*r* = 0.39) and excess mortality (*r* = 42) as well as with the higher impact of the pandemic (*r* = 0.21). The positive relationship between Uncertainty Avoidance correlated with COVID-19 deaths and excess mortality was also found in regression analyses. Interestingly, Uncertainty Avoidance also predicted the higher vaccination percentage indicating that people scoring high in Uncertainty Avoidance are more worried about the severe consequences of SARS-CoV-2 infection and rely on the vaccine than people scoring low in Uncertainty Avoidance. This is understandable because being vaccinated reduces the likelihood of adverse effects and, thus, reduces uncertainty. However, it is difficult to explain the positive correlation between Uncertainty Avoidance correlated with COVID-19 deaths and excess mortality since people with high Uncertainty Avoidance should welcome all measures (e.g., masks, lockdowns) to reduce the likelihood of SARS-CoV-2 infection. Uncertainty Avoidance, however, had almost a zero correlation (*r* = − 0.02) with the Stringency Index.

In the current study, Power Distance correlated significantly with excess mortality but not with COVID-19 deaths per capita, which might explain why earlier studies about Hofstede’s cultural dimensions have not reported any relationships between Power Distance and COVID-19 deaths. Power Distance also correlated positively with the impact of the COVID-19 pandemic score, indicating that high Power Distance countries suffered more from the pandemic than lower Power Distance countries. This result was also found in regression analysis. Besides, Power Distance was negatively related to GDP change during the pandemic in correlation and regression analyses indicating that high Power Distance countries suffered economically more from the pandemic than low Power Distance countries. Power Distance also correlated with low vaccination percentage, but this result was not significant in the regression analysis: Individualism and Uncertainty Avoidance were more important predictors in the model.

Hofstede’s “new” dimensions Short-Term/Long-Term Orientation and Indulgence/Resistance also were related to COVID-19 variables. Long-Term Orientation correlated/predicted negatively impact of the pandemic, meaning that Long-Term oriented countries were less negatively affected by the pandemic. Also, Indulgence/Resistance correlated negatively with excess mortality and with effects of the COVID-19 pandemic but positively with vaccination rate. Long-Term Orientation was also negatively related to Stringency index score, which means that long-term oriented countries were less likely to restrict citizen’s freedom. Oey and Rahardjo’s (2021) claim that the "combination of high individualism and indulgence leads the United States to selfishness and lack of concern for others' well-being" did not get support from the present study if we take the US as an example of high indulgence and Individualism since neither Individualism nor indulgence was positively related to COVID-19 mortality.

The findings of the current study based on the pandemic situation in July 2021 differed drastically from the earlier studies based on 2020 data. This underlines the nature of the COVID-19 pandemic and maybe of all pandemics in the past (Spanish flu in 1918) and in future. Meng studied the spread of the COVID-19 pandemic in G20 countries and found that the cases and deaths related to the COVID-19 pandemic had a nonlinear nature and convergence [58]. This means that results about socio-economic, cultural, and population correlates of the effects of the pandemic depend strongly on the sample of countries (e.g., high-income vs low-income) and the phase of the pandemic. The pandemics spread to different regions and countries at a different speed, which influences the correlations between socio-cultural factors and the outcome measures of the pandemic. The same applies to vaccinations and other countermeasures such as recommendations and restrictions: countries apply various policies and countermeasures (e.g., vaccinations) as responses in different stages. It is important to bear in mind that both the outcomes of the pandemic (infections, deaths) and especially countermeasures (e.g., lockdowns) influence people’s lifestyle and behaviour, which in turn, may be reflected in socio-economic (e.g., economic activity in a region) and population (e.g., obesity, alcohol use) indicators. In sum, the present study shows that the findings about socio-economic and cultural correlates of the pandemic outcomes (deaths, infections) depend on the stage of the pandemic and the countries studied. The final conclusions about the socio-cultural correlates of the COVID-19 pandemic can be drawn only after the pandemic is clearly over. In future studies taken place after the COVID-19 pandemic, the time, stage and spread of the pandemic, as well as the countermeasures (e.g., vaccinations), should be included in the panel or time-series analysis.

Another important question is how the underlying socio-economic, cultural, and population-related factors can be considered in battling against future pandemics. While the socio-economic and cultural factors might not be directly linked to pandemic mortality or infection rates, they can give important information for planning campaigns and interventions for changing attitudes. Since many of the countermeasures such as obligatory face mask use, social distancing and lockdowns, and vaccination intake are based on people’s readiness to accept and apply measures introduced by the health authorities, information campaigns should be designed according to the target population. In countries scoring high on individualism, for example, the messages should focus on individuals’ own vulnerabilities and benefits, while in collectivistic countries, the focus should be on an individual’s responsibility for others, i.e., family and the local community. Similarly, cultures scoring high on Uncertainty Avoidance may be more prone to conspiracy theories and less likely to trust governmental information. In this case, health campaigns could mainly focus on the trustworthiness of the information provided (e.g., safety and efficiency of the vaccine). These are just a few examples of how the present study's findings and future studies about culture and socio-economic factors in health behaviour can be used in future pandemics.

## Conclusions

The present study results somewhat differed from the earlier findings: Individualism was not related to COVID-19 deaths but instead was related to the low impact of the pandemic. Uncertainty Avoidance correlated with COVID-19 deaths, whereas Uncertainty Avoidance and Power Distance correlated significantly with excess mortality. In addition, countries with Long-Term Orientation were less impacted by the pandemic. In addition to these results about cultural dimensions and COVID-19 indexes, this study has other even more important messages. First, the COVID-19 pandemic reached different countries and regions at different times, which is clearly reflected in the results. The final conclusion about the role of socio-economic and cultural factors in managing the COVID-19 pandemic can be drawn only after the pandemic, not during the pandemic. Second, cultural dimensions were related to COVID-19 measures only when socio-economic indicators were not considered but lost their significance when socio-economic variables were entered into the models. This can mean that cultural dimensions influence the outcome variables via socio-economic and political factors. Further studies are needed to describe how the effects of the socio-economic and population factors mediate the effects of cultural values on the outcomes of the pandemic. Third, earlier studies have focused mainly on COVID-19 deaths. The present study shows that the impact of the COVID-19 pandemic is a complex phenomenon and cannot be reduced to the death rate. Other outcome variables such as excess mortality as well as the impact on the economy and citizens' freedom should be taken into account.

## Data Availability

Data used in the current study is available on reasonable request from the corresponding author.

## Abbreviations

BMI: Body-mass index
COVID-19: Coronavirus disease 2019
GDP: Gross domestic product
IVR: Indulgence
LTOWS: Long-term orientation
OECD: Organisation for Economic Co-operation and Development
PDI: Power distance
SARS-CoV-2: Severe acute respiratory syndrome coronavirus 2
UA: Uncertainty avoidance
WGI: Worldwide governance indicators
